# Inhibition of NKCC1 Modulates Alveolar Fluid Clearance and Inflammation in Ischemia-Reperfusion Lung Injury via TRAF6-Mediated Pathways

**DOI:** 10.3389/fimmu.2018.02049

**Published:** 2018-09-13

**Authors:** Chih-Hao Shen, Jr-Yu Lin, Yung-Lung Chang, Shu-Yu Wu, Chung-Kan Peng, Chin-Pyng Wu, Kun-Lun Huang

**Affiliations:** ^1^Division of Pulmonary and Critical Care, Department of Internal Medicine, Tri-Service General Hospital, Taipei, Taiwan; ^2^Graduate Institute of Medical Sciences, National Defense Medical Center, Taipei, Taiwan; ^3^Institute of Aerospace and Undersea Medicine, National Defense Medical Center, Taipei, Taiwan; ^4^Department of Biochemistry, National Defense Medical Center, Taipei, Taiwan; ^5^Department of Critical Care Medicine, Landseed Hospital, Taoyuan, Taiwan

**Keywords:** Na-K-2Cl cotransporter 1, bumetanide, ischemia-reperfusion, acute lung injury, epithelial sodium channel, p38 mitogen-activated protein kinase, tumor necrosis-associated factor 6, alveolar fluid clearance

## Abstract

**Background:** The expression of Na-K-2Cl cotransporter 1 (NKCC1) in the alveolar epithelium is responsible for fluid homeostasis in acute lung injury (ALI). Increasing evidence suggests that NKCC1 is associated with inflammation in ALI. We hypothesized that inhibiting NKCC1 would attenuate ALI after ischemia-reperfusion (IR) by modulating pathways that are mediated by tumor necrosis-associated factor 6 (TRAF6).

**Methods:** IR-ALI was induced by producing 30 min of ischemia followed by 90 min of reperfusion *in situ* in an isolated and perfused rat lung model. The rats were randomly allotted into four groups comprising two control groups and two IR groups with and without bumetanide. Alveolar fluid clearance (AFC) was measured for each group. Mouse alveolar MLE-12 cells were cultured in control and hypoxia-reoxygenation (HR) conditions with or without bumetanide. Flow cytometry and transwell monolayer permeability assay were carried out for each group.

**Results:** Bumetanide attenuated the activation of p-NKCC1 and lung edema after IR. In the HR model, bumetanide decreased the cellular volume and increased the transwell permeability. In contrast, bumetanide increased the expression of epithelial sodium channel (ENaC) via p38 mitogen-activated protein kinase (p38 MAPK), which attenuated the reduction of AFC after IR. Bumetanide also modulated lung inflammation via nuclear factor-κB (NF-κB). TRAF6, which is upstream of p38 MAPK and NF-κB, was attenuated by bumetanide after IR and HR.

**Conclusions:** Inhibition of NKCC1 by bumetanide reciprocally modulated epithelial p38 MAPK and NF-κB via TRAF6 in IR-ALI. This interaction attenuated the reduction of AFC via upregulating ENaC expression and reduced lung inflammation.

## Introduction

Acute lung injury (ALI) induced by ischemia-reperfusion (IR) results in severe hypoxemia in patients with lung transplantation, cardiopulmonary bypass, trauma, pulmonary embolism, and resuscitation for circulatory arrest. ALI also has high rates of mortality and morbidity (1, 2). IR causes the production of pro-inflammatory cytokines and impairs the integrity of the alveolar–capillary barrier. The process leads to increased microvascular permeability, the influx of protein-rich fluid, and the formation of lung edema (2–4).

Fluid hemostasis is important for ALI. Alveolar fluid secretion (AFS) involves chloride secretion in combination with secondary fluid flux into the alveolar space, and alveolar fluid clearance (AFC) refers to the capability of the alveolae to clear edema fluid (5). In severe ALI, pro-inflammatory cytokines result in both increased AFS and impaired AFC (6–8). Several transporters on the alveolar epithelium are involved in AFS and AFC, such as Na-K-2Cl cotransporter 1 (NKCC1), cystic fibrosis transmembrane conductance regulator (CFTR), and epithelial sodium channel (ENaC). ENaC participates in water reabsorption from the alveolar space in ALI (5, 8).

NKCC1 regulates intracellular volume by coupling the transport of sodium, chloride, and potassium, which create a driving force for water transport (9, 10). It is ubiquitously expressed in cells and serves various functions in different tissues (11–17). In alveolar epithelial cells, NKCC1 is mainly expressed in the basolateral membrane and impacts the generation of AFS. The expression of NKCC1 is upregulated in response to cardiogenic pulmonary edema and non-cardiogenic pulmonary edema induced by the administration of lipopolysaccharide (LPS) and leptospirosis infection (5, 18, 19). Inhibiting NKCC1 can attenuate cardiogenic pulmonary edema by decreasing AFS in the isolated perfused lungs of rats (20). In the absence of lysine kinase 4 (WNK4), which is upstream of NKCC1, AFC is also regulated by NKCC1 in mice with hyperoxia-induced lung injury (18). However, the exact mechanism of the modulation of AFC by NKCC1 remains unclear.

Increasing evidence suggests that the inhibition of NKCC1 may reciprocally attenuate inflammation in lung diseases. Inflammation caused by pulmonary bacterial infection was minimized in mice lacking NKCC1 (19). The inhalation of furosemide, an NKCC1 inhibitor, had an anti-inflammatory effect for asthma (21–23). Recently, we demonstrated that the functional reduction of NKCC1 by genetic or pharmacologic treatment suppressed nuclear factor-κB (NF-κB) and reduced the severity of IR-ALI in mice (24). The role of NKCC1 inhibition in attenuating lung inflammation in ALI warrants further investigation.

The activation of the Toll-like receptor (TLR) signaling pathway is involved in IR-ALI (25, 26). The adaptor proteins of TLRs, including myeloid differentiation primary response gene 88 (MyD88) and IL-1R-associated kinase 1/4 (IRAK1/4), form a complex with tumor necrosis factor-associated factor 6 (TRAF6) that participates in the transduction of upstream TLRs signals (27, 28). TRAF6 polyubiquitination can activate downstream pathways such as NF-κB and mitogen-activated protein kinases (MAPKs), which regulate apoptosis, modulate the production of pro-inflammatory cytokines, and exhibit various cellular functions in ALI (29–34). Hence, TRAF6 is speculated to have a role in the modulation of IR-ALI by NKCC1.

Bumetanide is a specific NKCC1 inhibitor that is approved by the United States Food and Drug Administration for clinical use as a potent loop diuretic (35). Alveolar NKCC1 is a potential target for therapeutic intervention, which means that bumetanide may exhibit extra-renal effects in ALI. The aim of this study is to investigate the role of inhibiting NKCC1 in IR-ALI *in vitro* and *ex vivo*.

## Methods

### Isolated perfused lung model in rats

Male Sprague-Dawley rats (350 ± 20 g) were purchased from BioLASCO Taiwan Co., Ltd. (Taipei, Taiwan) and cared for according to the guidelines of the National Institutes of Health (US NIH). The experiments were approved by the Institutional Animal Care and Use Committee of the National Defense Medical Center. The isolated and perfused rat lungs were prepared using previously described methods (36). Briefly, rats were anesthetized with intraperitoneal sodium pentobarbital (50 mg/kg), and tracheostomy was performed. The rats were then ventilated with humidified air containing 5% CO_2_ with a positive end-expiratory pressure of 1 cm H_2_O during the experiments. The ventilator settings also included a tidal volume of 3 mL and a frequency of 60 breaths/min.

After a median sternotomy, heparin (1 U/g of BW) was injected into the right ventricle, and 10 mL of blood was collected from the cardiac puncture. The isolated lung was perfused with 10 ml of collected blood and 10 mL of physiological salt solution containing 4% bovine serum albumin. An afferent cannula was inserted into the pulmonary artery, and an effluent cannula was inserted into the left atrium through the left ventricle to collect the effluent perfusate. The left atrial pressure, which represents the pulmonary venous pressure (PVP), was monitored from a side arm of the outflow cannula. The pulmonary arterial pressure (PAP) was monitored in the side arm of the inflow cannula. A roller pump was used to provide a constant flow rate of 7 mL/min. The isolated perfused lung *in situ* was placed on an electronic balance to record the changes in lung weight in real time.

### Experimental protocols

The rats were randomly assigned to four groups: a control group, control + bumetanide group, IR group, and IR + bumetanide group. Bumetanide was administered at the time of reperfusion via the perfusate. In the IR group, the lungs were subjected to ischemia by stopping ventilation and perfusion for 30 min and then reperfused and ventilated for 90 min after the ischemia. Three doses (35, 70, 140 μg/kg) of bumetanide were administrated in the preliminary studies. We found bumetanide 70 μg/kg had the best effect to decrease lung edema in IR-ALI *ex vivo* (Figure S1).

### Measurement of AFC

AFC was determined using an *in situ* rat-lung model, as described previously (37). Briefly, at the end of reperfusion, rat lungs were inflated with 100% oxygen at 7 cm H_2_O continuous positive airway pressure. Then, instillate containing fluorescein isothiocyanate (FITC)-labeled albumin (Sigma-Aldrich, St. Louis, MO) was delivered to the lungs over 1 min at 12.5 mL/kg of body weight of. An alveolar fluid sample (100 μL) was collected 1 min after instillation and 15 min later. The samples were centrifuged at 3,000 × g for 10 min, and the fluorescence activity in the supernatant was measured in duplicate. AFC was computed from the increase in alveolar fluid albumin concentration using the following equation:

$$\begin{array}{l} \text{AFC}=\left({\text{C}}_{\text{f}}-{\text{C}}_{\text{i}}\right)/{\text{C}}_{\text{f}}\times 100, \end{array}$$

where C_i_ and C_f_ represent the initial and final concentrations of FITC-albumin in the aspirate at 1 and 15 min, respectively, as assessed by the fluorescence activity measurements.

### MLE-12 cell culture

The mouse lung epithelial cell line MLE-12 (CRL-2110) was obtained from the American Type Culture Collection (ATCC, Manassas, VA, USA). The cells were cultured in DMEM/F-12 media (Biological Industries, Beth Haemek, Israel), which contained 2% fetal bovine serum (FBS; Invitrogen, Carlsbad, CA, USA) and other relevant supplements: 5 μg/ml of insulin,10 μg/ml of transferrin, 2 mM of L-glutamine, 10 mM of HEPES (Biological Industries, Beth Haemek, Israel), 30 nM of sodium selenite, 10 nM of hydrocortisone, and 10 nM of β-estradiol (Sigma-Aldrich, St. Louis, MO, USA) at 37°C in a humidified atmosphere containing 5% CO_2_. The cells were passaged at approximately 80% confluence by incubation with 0.25% trypsin-EDTA. For the experiments, the cells were seeded in a six-well plate at a density of 3.5 × 10^4^ cells/cm^2^ and cultured for 24 h under the same conditions for expansion.

### Hypoxia-reoxygenation model in MLE-12 cells

The hypoxia-reoxygenation (HR) cells were prepared using previously described methods (38). After 24 h of incubation, the cells were pretreated with vehicle, bumetanide (10, 20, 40-μM), or p38 MAPK inhibitor BIRB-796 (10-μM; Enzo Life Sciences, Inc., San Diego, CA, USA). After 30 min of treatment, the cells were subjected to 24 h of hypoxia (5% CO_2_, 1% O_2_, and 94% N_2_), followed by reoxygenation (1, 2, 4 h; 5% CO_2_ in ambient air) at 37°C using the CO2/Tri-Gas Incubator (ASTEL).

### Bronchoalveolar lavage fluid protein concentration and perfusate tumor necrosis factor-α level

At the end of the experiment, bronchoalveolar lavage fluid (BALF) was obtained by lavaging the left lung twice with 2.5 mL of saline. The fluid was then immediately centrifuged at 200 × g for 10 min to remove all cells and cellular debris. The protein levels were measured using a Pierce™ BCA protein assay kit (Thermo Fisher Scientific). The tumor necrosis factor-α (TNF-α) in the perfusate was measured using a commercially available ELISA kit (R&D Systems Inc., Minneapolis, MN, USA).

### Immunoblotting

The lysates (30 μg/lane) of lung and cell culture protein were separated by sodium dodecyl sulfate–polyacrylamide gel electrophoresis (SDS-PAGE) and transferred to a polyvinylidene fluoride membrane (Millipore). The blots were incubated overnight with primary antibodies anti-α-ENaC, anti-TRAF6 (1:200, Santa Cruz Biotechnology, USA), anti-phosphorylated-p38, anti-p38, anti-phosphorylated-Erk, anti-Erk, anti-phosphorylated-JNK, anti-JNK, anti-phosphorylated-NF-κB p65, anti-IκB-α, anti-TATA (1:1,000, Cell Signaling Technology, USA), and anti-β-actin (1:10,000, Sigma Chemical Company, USA). The anti-phosphorylated NKCC1 and anti-NKCC1 antibodies were custom made by GeneTex.

### Immunohistochemical analyses

Immunohistochemical staining was performed to identify TRAF6. In brief, formalin-fixed paraffin lung tissue sections were de-paraffinized and pretreated for antigen retrieval. The endogenous peroxidase was quenched using 3% H_2_O_2_ and 100% methanol for 15 min. The lung sections were immunostained with the primary antibody overnight. The slides were then washed twice and incubated with horseradish peroxidase-conjugated secondary antibody (Nichirei Corporation, Tokyo, Japan) for 30 min. The horseradish peroxidase was visualized after a chromogenic reaction with diaminobenzidine for 3 min, and the lung tissue sections were counterstained with hematoxylin.

### Histopathology

The numbers of polymorphonuclear neutrophils and lung injury score in the lung tissue were analyzed. In brief, the lung tissues were fixed, sectioned, and stained with eosin and hematoxylin. Morphological examinations were performed using light microscopy. A minimum of 10 randomly selected fields were examined for neutrophil infiltration in the airspace or vessel wall. The thickening of the alveolar wall was also observed.

Next, the lung damage was scored as follows using a four-point scale: none (0), mild (1), moderate (2), or severe (3). The scoring was performed by two pathologists who were blinded to the experimental conditions. The two resulting scores were summed to represent the lung injury score.

### Immunofluorescence staining

Immunofluorescence staining was performed with a previously described method (39). The customized phosphorylated-NKCC1 antibody was used for immunofluorescent labeling overnight at 4°C and then incubated for 1 h at room temperature with chicken anti-rabbit IgG-fluoresce in isothiocyanate (Santa Cruz Biotechnology, USA) as the secondary antibody. After washing, the slides were mounted with VECTASHIELD Antifade Mounting Medium with DAPI. Images were obtained using a DeltaVision system (Applied Precision) comprising a wide-field inverted microscope (model IX-71; Olympus) with ×60/1.42 Plan Apo N or ×100/1.40 Super-Plan APO objectives. Images were captured using a CCD camera (Coolsnap HQ2; Photometrics) and Softworx analysis software (GE Healthcare).

### Flow cytometry

Flow cytometry was performed using a FACSVerse flow cytometer with FACSuite software (BD Biosciences, USA). For cell size measurements, cells were harvested at 1 h after reoxygenation, centrifuged at 500 × g for 5 min, and resuspended in 200 μl containing 2 × 10^6^ cells. The cells were then incubated with 10 μl of 7-amino-actinomycin D (7-AAD) staining medium (BD Biosciences) for 15 min. Doublets and debris were excluded from the data.

The forward scatter was evaluated, and experiments to analyze the cell size were performed on the same day to ensure that the voltage settings were consistent for accurate comparison. To detect the expression of ion transporters, cells were immunoreacted with the customized primary antibody anti-phosphorylated NKCC1 by GeneTexor anti-α-ENaC (Santa Cruz Biotechnology) and chicken anti-rabbit IgG-fluoresce secondary antibody in isothiocyanate (Santa Cruz Biotechnology). The analyses were performed with FACSuite software.

### Quantitative real-time PCR

The total RNA was isolated using an RNA-spin total RNA extraction kit (Intron Biotechnology, Korea) according to the manufacturer's instructions. The synthesis of cDNA was performed with 2 μg of RNA using a High-Capacity cDNA Archive Kit (Applied Biosystems, Foster City, CA, USA). Quantitative real-time PCR was performed for TRAF6 and GAPDH using TaqMan assays (Applied Biosystems, Foster City, CA, USA). Each sample was analyzed in triplicate on a 96-well plate, which was centrifuged briefly and placed in a ViiA 7 qRT-PCR machine (Thermo Fisher Scientific, Waltham, MA, USA). The analysis was performed using the following program: 2 min at 50°C, 10 min at 95°C, and 40 cycles of 15 s at 95°C and 1 min at 60°C. The relative gene expression was calculated using the 2-ΔΔCT method.

### Transwell monolayer permeability assay

To measure the paracellular permeability of epithelial cells, MLE-12 cells were grown as a monolayer in 6.5-mm-diameter transwell filter inserts with a pore size if 3.0 μm (Corning Life Sciences, Lowell, MA) and placed in 24-well tissue culture plates. The cells were examined for consistency, confluence, and integrity of the monolayer. After 24 h of exposure to hypoxia, the medium of the upper chamber was replaced with medium containing albumin-fluorescein isothiocyanate (2 mg/mL) and left to reoxygenate for 3 h. Samples (100 μL) were collected from the lower chambers and analyzed for fluorescein isothiocyanate intensity using a fluorometric plate reader with an excitation of 494 nm and emission at 520 nm. The data were calculated as percentages of the control values.

### Statistical analysis

The data are expressed as the mean ± SD. Comparison among groups was performed using one-way or two-way repeated-measures analysis of variance (ANOVA), followed by a *post-hoc* comparison using the Newman-Keuls test. Statistical significance was defined by *p* < 0.05.

## Results

### Bumetanide attenuates IR-induced lung edema *ex vivo*

**Figure 1:**
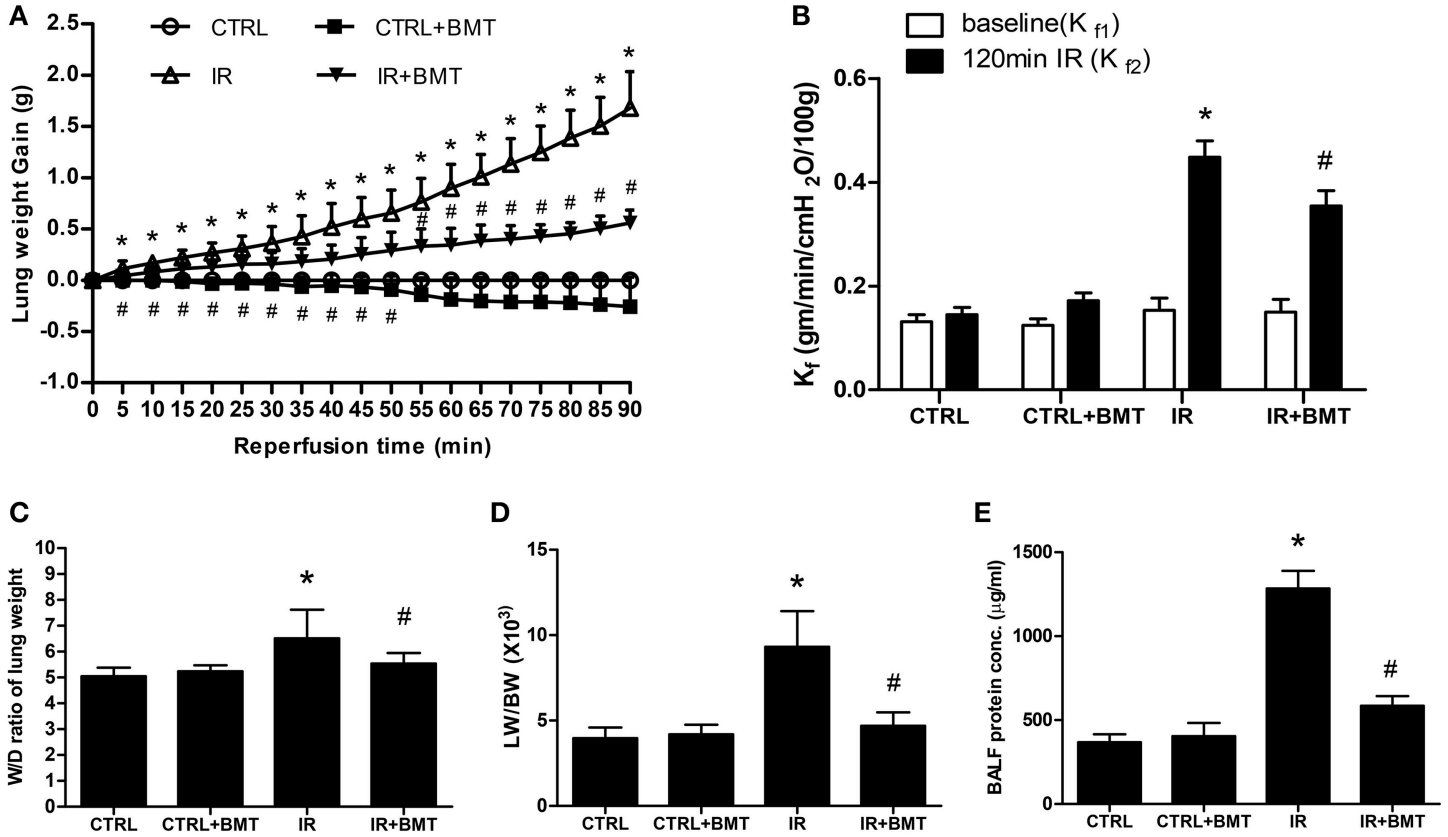
Effects of BMT on lung edema. **(A)** Lung weight gain, **(B)** pulmonary microvascular permeability (Kf), **(C)** lung wet/dry (W/D) weight ratios, **(D)** lung weight/body weight (LW/BW), and **(E)** protein concentration in bronchoalveolar lavage fluid (BALF). The increase of these parameters in the ischemia–reperfusion (IR) group was significantly attenuated by treatment with BMT. BMT, bumetanide 70 μg/kg; CTRL, control. Data are expressed as the mean ± SD (*n* = 6 per group). *P < 0.05 compared with the control group; ^#^*P* < 0.05 compared with the IR group.

Bumetanide significantly attenuated the increases in lung weight gain, vascular filtration coefficient (Kf), the ratio of wet weight to dry weight ratio (W/D) and the ratio of lung weight to body weight (LW/BW) in the IR group (Figures 1A–D). These results suggest that bumetanide decreases lung edema in IR-ALI *ex vivo*. The protein concentration in BALF was also measured as an indicator of dysfunction in the alveolar–capillary barrier, and bumetanide significantly decreased the elevated protein concentration in the IR group (Figure 1E).

### Bumetanide attenuates the activation of epithelial phosphorylated-NKCC1 in IR-ALI

**Figure 2:**
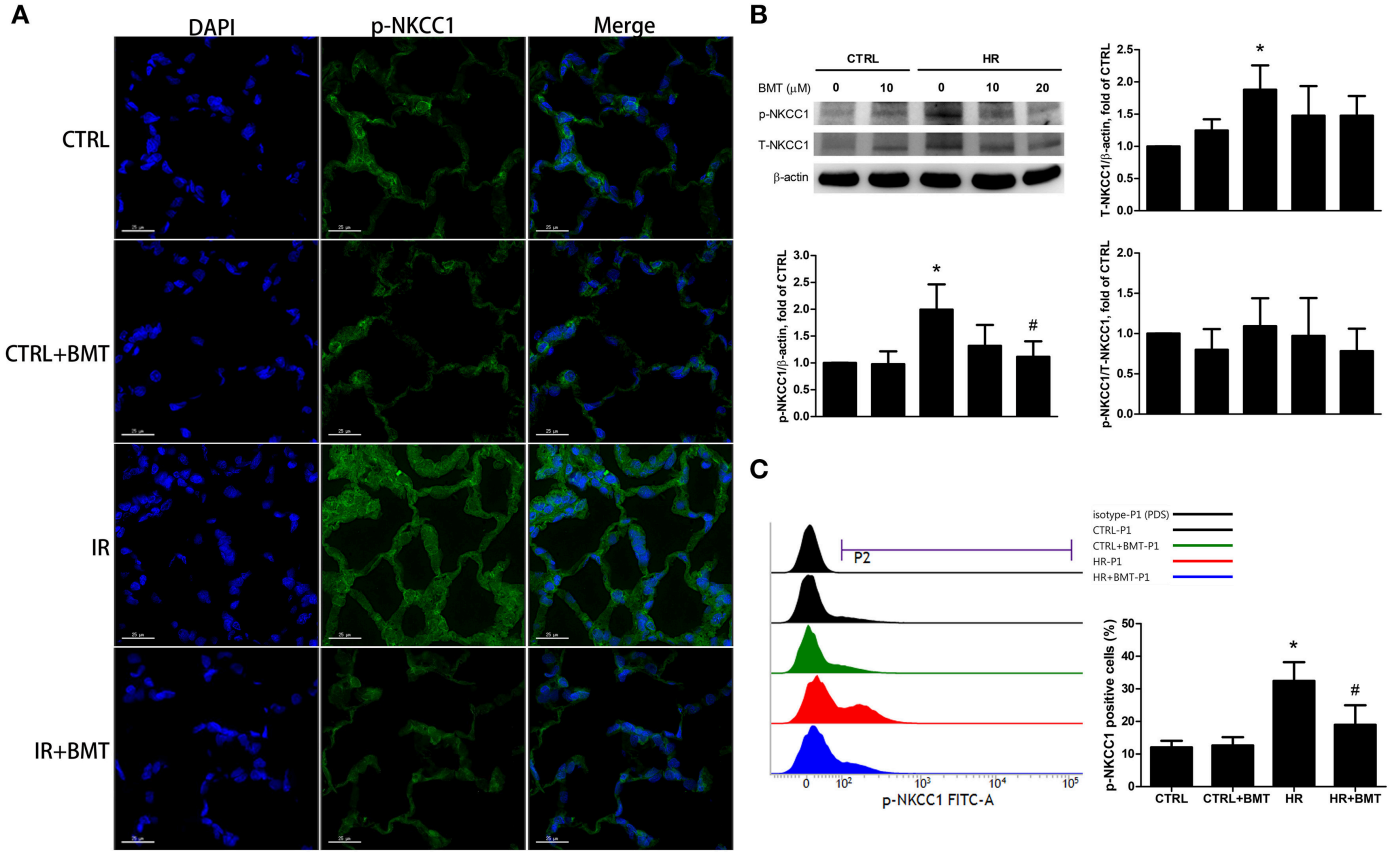
Expressions of p-NKCC1 in rat lung tissues and MLE-12 cells. **(A)** Representative images of p-NKCC1 immunofluorescence staining (FITC-labeled green; original magnification ×400) of rat lung. Nuclei were counterstained with DAPI (blue). **(B)** T-NKCC1 and p-NKCC1 expressions in MLE-12 cells determined by western blot analysis (*n* = 5 per group). **(C)** Histogram of p-NKCC1 determined by flow cytometry in MLE-12 cells (*n* = 3 per group). BMT attenuated the activation of epithelial p-NKCC1 in IR-ALI. BMT, bumetanide 20-μM in HR model and 70 μg/kg in IR model; CTRL, control. Data are expressed as the means ± SD. *P < 0.05 compared with the control group; ^#^*P* < 0.05 compared with the HR group.

The immunofluorescence results showed that epithelial phosphorylated-NKCC1 (p-NKCC1) increased markedly in the IR group, and the increase was significantly less pronounced in the IR + bumetanide group (Figure 2A). The p-NKCC1 was activated in the HR model in comparison with the control group (Figure 2B). A 20-μM concentration of bumetanide was sufficient to suppress the expression. Flow cytometry demonstrated the same effect of bumetanide on p-NKCC1 after HR (Figure 2C). To demonstrate the possible mechanisms underlying bumetanide effects on NKCC1 expression, we performed quantitative real-time PCR for NKCC1 using TaqMan assays. Similarly, a non-significant decrease of NKCC1 mRNA expression was found in HR + bumetanide group in comparison to the HR group (Figure S2). These results suggest that IR activates p-NKCC1 in the alveolar epithelium and that bumetanide inhibits p-NKCC1 in IR-ALI.

### Bumetanide increases cellular volume and decreases paracellular permeability of alveolar epithelium in HR model

**Figure 3:**
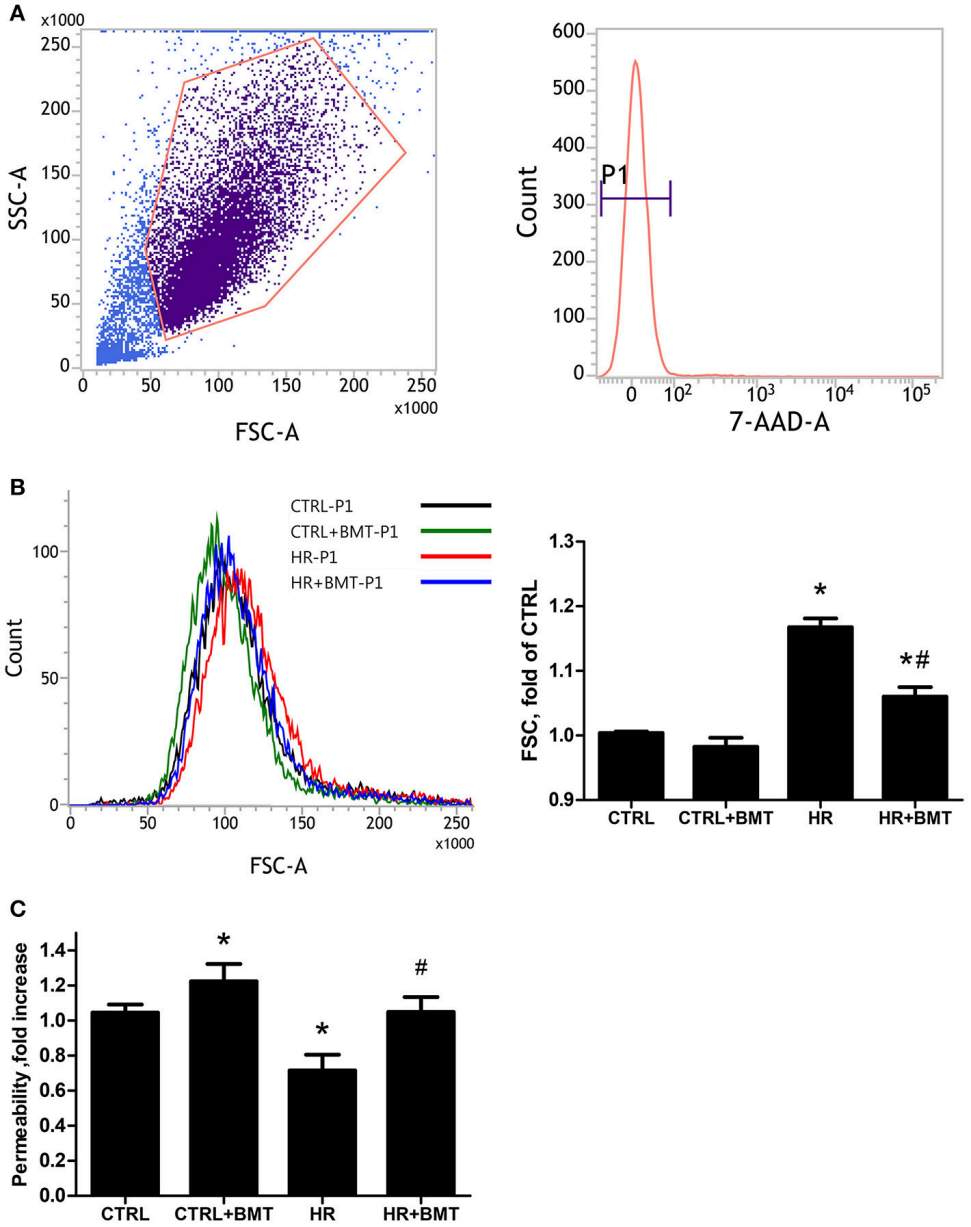
Cell size and paracellular permeability in MLE-12 cells. **(A)** Representative flow cytometry dot plots showing the gating strategy for identification of side scatter (SSC) and forward scatter (FSC) in MLE-12 cells, followed by hierarchical subgating according to negative 7-Amino-Actinomycin D (7-AAD) staining (P1). **(B)** Histograms of cell sizes measured by forward scatter (FSC) after 1 h of reoxygenation. Data are expressed as the means ± SD from independent experiments (*n* = 3 per group). **(C)** Paracellular permeability assay using MLE-12 cells cultured with FITC-dextran (4 kDa, 2 mg/ml). Data are expressed as the mean ± SD (*n* = 9 per group). BMT, bumetanide 20-μM; CTRL, control. *P < 0.05 compared with the control group; ^#^*P* < 0.05 compared with the HR group.

We used the forward scatter (FSC) to represent the cellular volume and transwell monolayer to examine the paracellular permeability of MLE-12 cells. There was a significant increase in FSC in the HR group, which was attenuated by bumetanide (Figures 3A,B). However, paracellular permeability decreased in the HR group and was increased by treatment with bumetanide (Figure 3C). These results indicate that bumetanide attenuates IR-induced lung edema, which may not result from the change in cellular volume induced by NKCC1.

### Bumetanide increases epithelial α-ENaC expression and attenuates the reduction of AFC in IR-ALI

**Figure 4:**
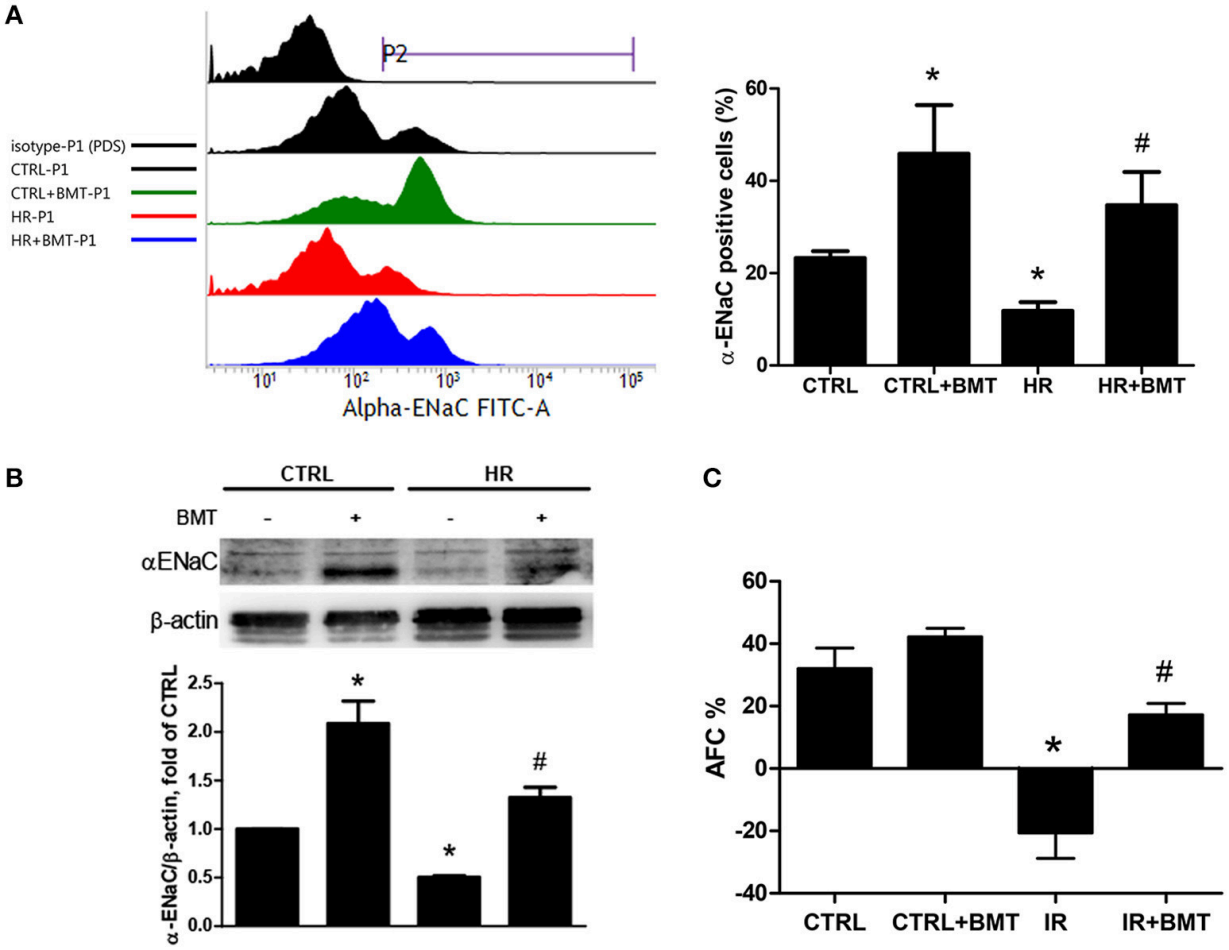
α-ENaC expression and alveolar fluid clearance (AFC). **(A)** Histogram show the expression of α-ENaC on the MLE-12 cells (P2). Cells were gated according to negative 7-Amino-Actinomycin D (7-AAD) staining (P1; *n* = 3 per group). **(B)** α-ENaC levels in MLE-12 cells determined by western blot analysis (*n* = 5 per group). **(C)** Effects of AFC in response to BMT treatment (*n* = 5 per group). BMT, bumetanide 20-μM in HR model and 70 μg/kg in IR model; CTRL, control. Data are expressed as the means ± SD. *P < 0.05 compared with the control group; ^#^*P* < 0.05 compared with the HR group.

Flow cytometry was performed to clarify the effect of NKCC1 on α-ENaC after HR. The ratio of α-ENaC-positive cells decreased after HR and was restored by bumetanide (Figure 4A). The protein expression of α-ENaC decreased significantly after HR, which was increased by bumetanide (Figure 4B). Similar results were found in rats with IR-ALI (Figure S3A). Because α-ENaC participates in water reabsorption from the alveolar space in ALI, AFC was measured. In the IR group, AFC decreased markedly. The decrease in AFC was restored in the IR + bumetanide group (Figure 4C).

### Bumetanide modulates epithelial α-ENaC via p38 MAPK in IR and HR models

**Figure 5:**
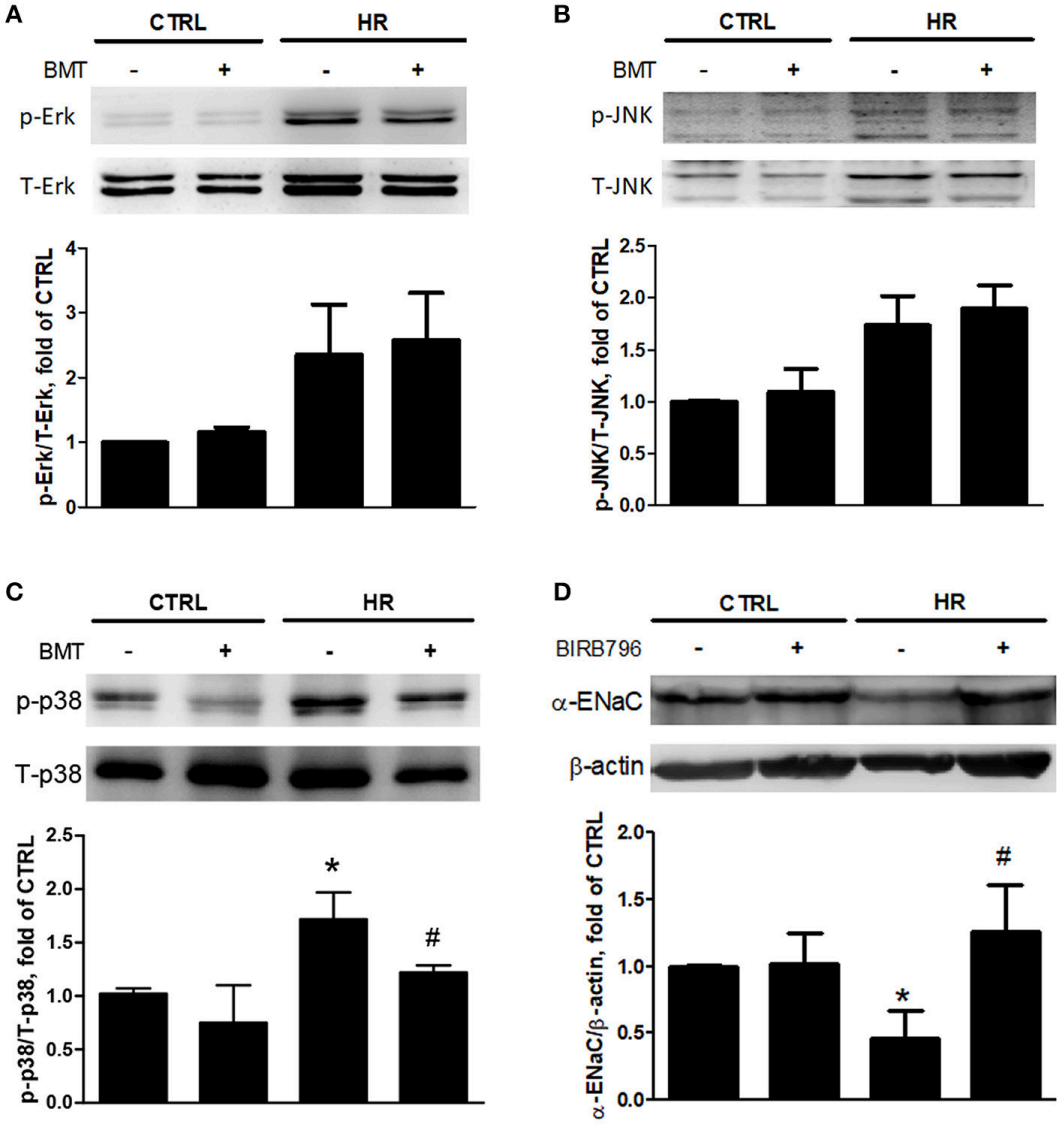
Expressions of MAPKs and α-ENaC in MLE-12 cells. **(A)** Total Erk MAPK (T-Erk) and phosphorylated Erk MAPK (p-Erk), **(B)** total JNK MAPK (T-JNK) and phosphorylated JNK MAPK (p-JNK), **(C)** total p38 MAPK (T-p38) and phosphorylated p38 MAPK (p-p38) after HR treated with BMT. **(D)** α-ENaC expression after HR treated by p38 MAPK inhibitor, BIRB-796 10 μM. BMT, bumetanide 20-μM; CTRL, control. Data are expressed as the mean ± SD (*n* = 5 per group). *P < 0.05 compared with the control group; ^#^*P* < 0.05 compared with the HR group.

To determine how epithelial NKCC1 modulates IR-ALI, three isoforms of MAPKs, including p38, Erk and JNK, were measured in the HR model. The expressions of phosphorylated -p38 MAPK (p-p38), phosphorylated -Erk MAPK (p-Erk) and phosphorylated -JNK MAPK (p-JNK) were activated after HR. Only p-p38 was inhibited by bumetanide (Figures 5A–C). The dose- and time- dependency of bumetanide effects on T-p38 and p-p38 were shown in Figure S4. The protein expression of p-p38 increased significantly in rats with IR-ALI, which was decreased by bumetanide (Figure S3B). To assess the role of p38 MAPK in modulating the α-ENaC expression, MLE-12 cells were pretreated with p38 MAPK inhibitor (BIRB-796) 10 μM, followed by HR. HR resulted in lower α-ENaC expression, which was restored by Birb-796 (Figure 5D). Similar results were found in rats pretreated with BIRB-796 0.3 mg/kg in IR-ALI (Figure S3C).

### Epithelial NF-κB and inflammation are modulated by bumetanide in IR-ALI

**Figure 6:**
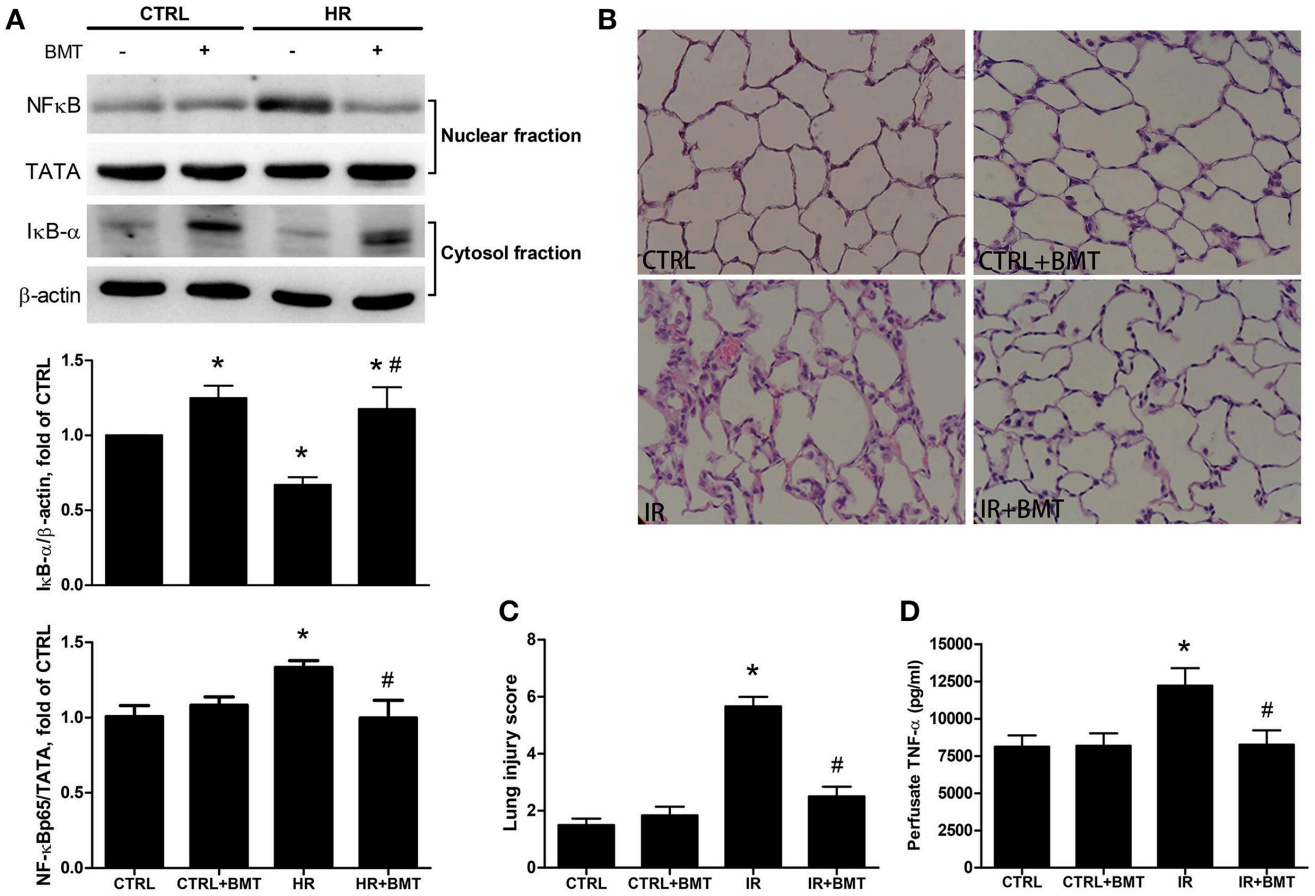
Expressions of epithelial NF-κB and inflammatory parameters in IR-ALI. **(A)** IκB-α and nuclear phosphorylated NF-κB p65 levels in MLE-12 cells treated with BMT. **(B)** Hematoxylin and eosin staining for lung tissue (200× magnification). **(C)** Lung injury score in IR model. **(D)** TNF-α level in the perfusate in IR model. TATA and β-actin served as loading controls for nuclear and cytoplasmic proteins, respectively. BMT, bumetanide 20-μM in HR model and 70 μg/kg in IR model; CTRL, control. Data are expressed as the means ± SD (*n* = 5 per group). *P < 0.05 compared with the control group; ^#^*P* < 0.05 compared with the HR group.

Compared with the control group, western blot analysis indicated higher nuclear levels of NF-κB p65 in the HR group, whereas the levels of IκB-α were significantly suppressed. Bumetanide restored the suppressed IκB-α levels and reduced the nuclear NF-κB p65 levels after reoxygenation (Figure 6A). The dose- and time-dependency of bumetanide effects on NF-κB p65 and IκB-α were shown in Figure S4. Histological evaluation of lung tissues showed indicated a high lung injury score after IR (Figures 6B,C). IR significantly increased TNF-α levels in the perfusate compared with those in the control group. However, bumetanide significantly attenuated these increases (Figure 6D). Compared with the IR group, these indices of lung inflammation were significantly lower in the IR + bumetanide group (Figures 6B–D). These results show that bumetanide can inhibit epithelial inflammation via NF-κB in IR-ALI.

### Epithelial TRAF6 is attenuated by bumetanide in IR-ALI

**Figure 7:**
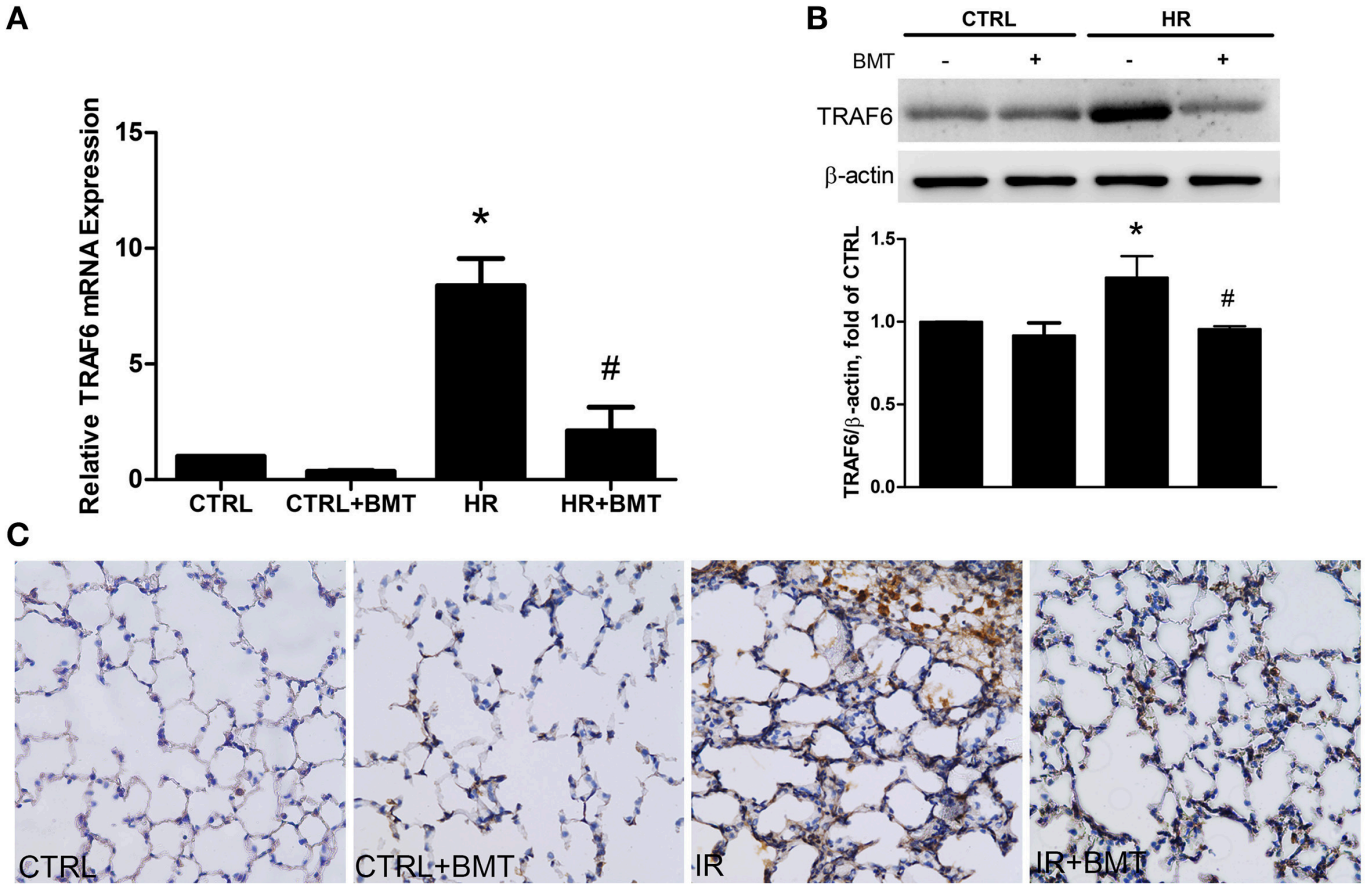
TRAF6 expression in IR and HR models. **(A)** TRAF6 mRNA levels in MLE-12 cells. **(B)** Protein levels in MLE-12 cells determined by western blot analysis. **(C)** Immunohistochemical staining for TRAF6 in lung tissue (200× magnification). In the IR and HR groups, TRAF6 expressions were significantly increased and decreased after BMT treatment, respectively. BMT, bumetanide 20-μM in HR model and 70 μg/kg in IR model. All values are expressed as the means ± SD (*n* = 5 per group). *p < 0.05 compared with the control group; ^#^*P* < 0.05 compared with the HR group.

TRAF6 is upstream of p38 MAPK and NF-κB. In the bumetanide + HR group, TRAF6 mRNA and protein expression were significantly decreased in comparison to the HR group (Figures 7A,B). Immunohistochemical staining indicated that the expression of TRAF6 in lung tissue was significantly higher in the IR group than in the control group and was significantly decreased by bumetanide treatment (Figure 7C).

## Discussion

**Figure 8:**
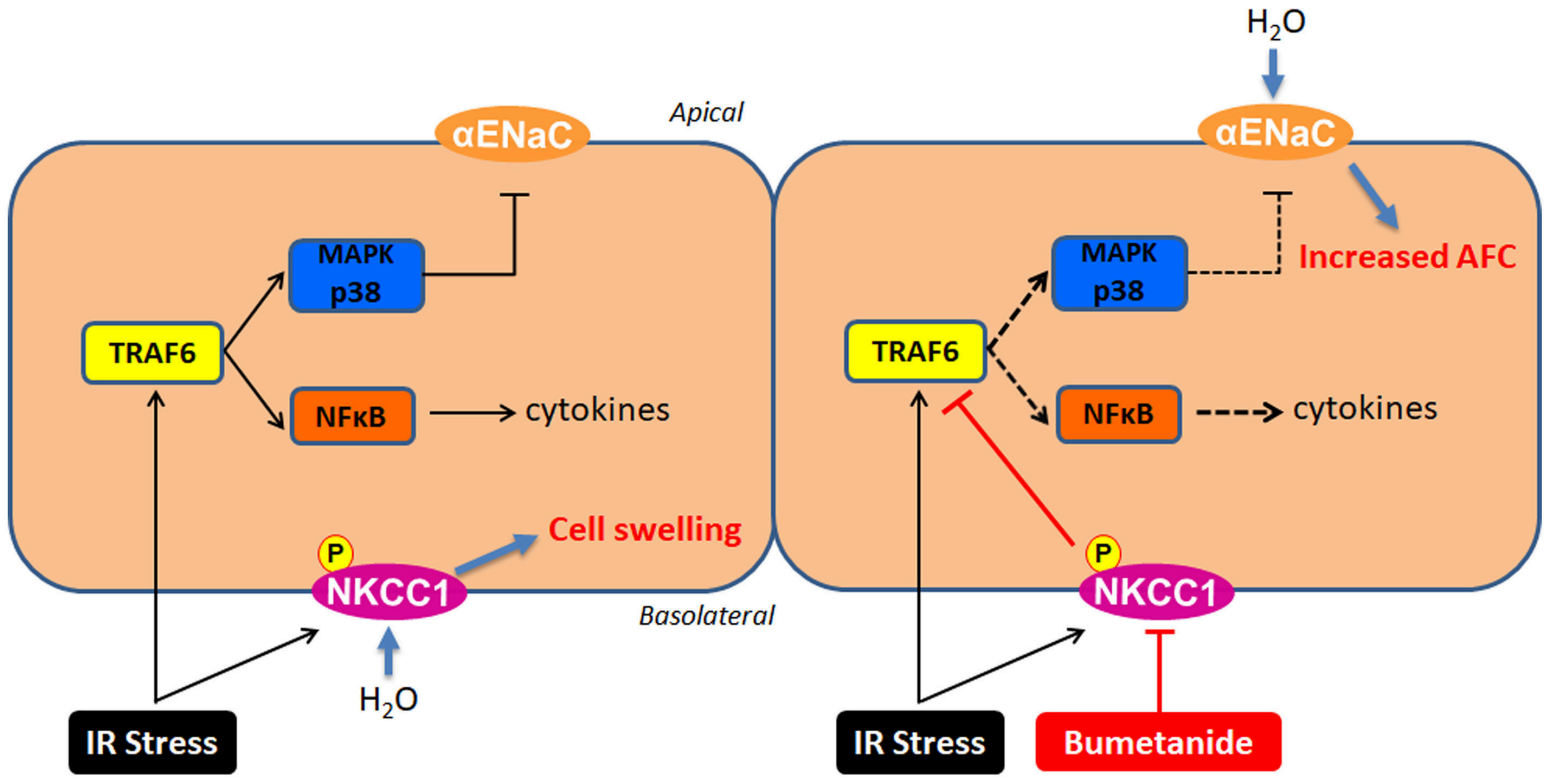
The mechanisms of NKCC1 modulating alveolar fluid clearance and inflammation in IR-ALI. IR stress causes phosphorylation of NKCC1 and activation of TRAF6, which result in cell swelling and inflammation of alveolar epithelium. Inhibition of NKCC1 by bumetanide reciprocally modulates epithelial TRAF6 expression. This interaction suppresses downstream p38 MAPK and NF-κB pathways, which attenuates the reduction of AFC via upregulating α-ENaC expression and reduces the alveolar inflammation.

We have presented the first research showing the mechanism of NKCC1 inhibition protecting the alveolar epithelium from IR-ALI with both *ex vivo* and *in vitro* experiments. The NKCC1 inhibitor bumetanide modulated p38 MAPK and α-ENaC after IR and compensated for the reduction in AFC. In addition, NF-κB was modulated by bumetanide, which attenuated lung inflammation. These mechanisms were mediated by suppressing TRAF6 expression (Figure 8).

In the present study, rats were used to obtain an isolated and perfused lung model *in situ*. Therefore, the diuretic effect of bumetanide toward lung edema was limited. Additionally, the *in vitro* HR model of mouse alveolar epithelial MLE-12 cells represented the condition of bumetanide acting in the absence of disturbance by pro-inflammatory cytokines released from alveolar macrophages, circulating monocytes, lymphocytes, and neutrophils. Bumetanide suppressed the expression of TRAF6 and TRAF6-mediated pathways in the HR model, which suggests that inhibiting NKCC1 may directly modulate the inflammation of alveolar epithelium in IR-ALI.

We found that bumetanide suppressed TRAF6 expression and attenuated the downstream inflammation. TLRs play a central role in innate immunity, which recognizes molecular patterns associated with both pathogens and damage. TLR2 and TLR4 may be activated by endogenous proteins that are released from tissues damaged by IR, such as low-molecular-weight hyaluronic acid, fibronectin, heat shock protein 70, and heparin sulfate, thus increasing TRAF6 expression (40, 41). A previous study showed that the down-regulation of TRAF6 is associated with the progression of acute pancreatitis with a complication of lung injury in mice (42). However, it remains unclear whether the TRAF6-mediated pathway is the only mechanism or the primary mechanism of mediating the effects of NKCC1.

Bumetanide lessened the reduction of AFC after IR. The activation of NKCC1 is known to mediate reversed transepithelial ion transport and drive active AFS in ALI (8). AFC was calculated from the increase in alveolar fluid albumin at the end of IR. The increased AFC in the IR + bumetanide group may be contributed by the attenuated AFS resulting from NKCC1 inhibition. In the same manner, the results showed that NKCC1 inhibition upregulated α-ENaC expression, which may also enhance alveolar fluid absorption. We found that bumetanide suppressed p38 MAPK and increased the expression of α-ENaC. Previous studies have shown that diverse extracellular stimuli, including IR, can trigger a stress-regulated protein kinase cascade that culminates in the activation of p38 MAPK through phosphorylation (43). Furthermore, pro-inflammatory cytokines may contribute to alveolar edema in ALI through the p38 MAPK-dependent inhibition of α-ENaC (44–46).

In the present study, bumetanide increased epithelial α-ENaC expression in basal condition. It is of interest that AFC and parameters of lung edema (lung weight gain, the ratio of wet weight to dry weight ratio and the ratio of lung weight to body weight) in basal condition were not affected by bumetanide as those recorded in IR condition (Figures 1, 4). A previous study for cardiogenic lung edema showed that NKCC1 inhibitors blocked AFS induced by elevated hydrostatic pressure. Similarly, in basal condition with normal hydrostatic pressure, NKCC1 inhibitors didn't alter the wet weight to dry weight ratio (20). We propose the possible mechanism that determines these findings. In addition to NKCC1 and ENaC, CFTR is also involved in the fluid homeostasis of alveolar epithelium. In the intact lung, hyperactivity of ENaC can be regulated by increased CFTR activity in airway epithelial cells (47, 48). Although bumetanide increases α-ENaC expression, activity of α-ENaC may be modulated by CFTR in basal condition. In ALI, by contrast, expression and function of CFTR are suppressed by released cytokines (8). Therefore, α-ENaC expression and AFC facilitated by bumetanide may be free from the influence of CFTR. Studies to investigate this field may be warranted in the future.

Bumetanide also suppressed NF-κB, which is also downstream of the pathway of TRAF6 in IR-ALI. The activation of NF-κB was followed by the production of TNF-α. TNF-α is an important pro-inflammatory cytokine that is produced by a composite of vascular endothelium, alveolar epithelium, and inflammatory cells, and its levels in the perfusate represent the local generation of TNF-α at the end of reperfusion (49, 50). Bumetanide modulated the production of TNF-α and the severity of ALI by the suppression of NF-κB. The present findings reinforce that TRAF6 plays a crucial role in the modulation of responses in IR-ALI by NKCC1.

The results showed that IR increased microvascular permeability, which was attenuated by bumetanide. Pulmonary microvascular permeability is the standard tool for the assessment of IR-induced lung injury. Nguyen et al. found that lung inflammation after infection with *Klebsiella pneumoniae* was minimized in mice lacking NKCC1 (19). Matthay et al. hypothesized that a lack of NKCC1 reduces the amounts of sodium and water that enter into endothelial and epithelial cells and that relatively normal cell shape would facilitate neutrophil transmigration and bacterial killing (51). Interestingly, the study also indicated that microvascular permeability after exposure to LPS was significantly lower in NKCC1 knockout mice than in wild-type mice. This observation is consistent with the findings in the present study.

It is therefore of interest to determine whether NKCC1 expression modulates the change in microvascular permeability by changing cell volume. The present study showed that bumetanide-treated epithelial cells had lower cell volume after HR, which correlated with greater paracellular permeability. In the lung, fluid flux into the adjacent interstitial space is regulated by gradients for hydrostatic forces and protein concentrations, as well as by permeability of the pulmonary barrier to water. These findings highlight that the change in epithelial cell volume may not determine microvascular permeability in the modulation of IR-ALI by NKCC1.

In the present study, we found that p-NKCC1 activity was inhibited significantly after bumetanide 20-μM treatment in HR, and a non-significant attenuation was found in bumetanide toward the mRNA and protein expression of T-NKCC1 (Figures 2, S2). In the model of rat traumatic brain injury, bumetanide suppressed the expression of T-NKCC1 in hippocampal neurons (52). On the other hand, in a humanglioma cells invasion study, the expression of p-NKCC1, not T-NKCC1, was apparently lower in the bumetanide-treated group compared to the control group (53). The role of bumetanide in inactivating NKCC1 may be more complex and needs to be elucidated by further studies.

For patients with edema associated with congestive heart failure, hepatic and renal disease, intravenous bumetanide 0.5–2 mg produces a rapid and marked diuresis without adverse effects. Overdose may lead to acute electrolyte depletion, reduction of blood volume and circulatory collapse with a possibility of vascular thrombosis and embolism (54). In the present study, the dose of bumetanide for rats was 70 μg/kg. To exchange doses between rat and human by allometric scaling, the human equivalent dose of bumetanide is 0.77 mg for a 60 kg human (55). It is less likely to cause adverse effects in this starting dose to treat patients with IR-ALI. Although the present study does not itself focus on clinical application, it is essential and the basis for the following translational studies.

The present study has two limitations in relation to the role of leukocytes and endothelium after IR. First, IR-ALI is a complex pathogenic condition that involves biochemical, cellular, and molecular alterations. Studies have shown that furosemide, another NKCC1 inhibitor, has an anti-inflammatory effect through the repression of inflammatory cytokines from peripheral mononuclear cells (16, 56). The roles of NKCC1 in neutrophils, macrophages, and lymphocytes are not well-known. Second, previous studies have shown that NKCC1 serves to regulate cell volume of endothelial cells. In vascular endothelium of brain, NKCC1 functions in the maintenance of a selective permeability barrier, as well as in the preservation of endothelial homeostasis in the face of fluctuating osmotic conditions (11, 57, 58). Although the present study demonstrated a relationship between epithelial NKCC1 and the alveolar-capillary permeability regardless of cell volume change in IR-ALI, the role of NKCC1 in the endothelial barrier of pulmonary vessels remains undetermined. Additionally, crosstalk between epithelium, endothelium and leukocytes is necessary to elucidate. A co-culture system in HR model may be warranted for identifying these interactions.

In conclusion, the inhibition of NKCC1 by bumetanide reciprocally modulated epithelial p38 MAPK and NF-κB via TRAF6 in IR-ALI. This interaction attenuated the reduction of AFC via upregulating α-ENaC expression and reduced lung inflammation. Clinical studies are warranted to determine the therapeutic effects of NKCC1 in IR-ALI in humans.

## Author contributions

C-HS, J-YL, C-PW, and K-LH conceived and designed the experiments. C-HS, J-YL, and S-YW performed the experiments. C-HS, J-YL, C-PW, and K-LH analyzed the data. Y-LC, S-YW, and C-KP contributed reagents, materials, analysis tools. C-HS wrote the paper.

### Conflict of interest statement

The authors declare that the research was conducted in the absence of any commercial or financial relationships that could be construed as a potential conflict of interest.

## Supplementary material

Figure S1 Effects of different doses of BMT on lung edema. **(A)** Lung weight/body weight (LW/BW), **(B)** lung wet/dry (W/D) weight ratios, and **(C)** pulmonary microvascular permeability (Kf). The increase of Kf in the ischemia–reperfusion (IR) group was significantly attenuated by treatment with BMT 70 μg/kg. BMT: bumetanide. CTRL: control. Data are expressed as the mean ± SD (*n* = 3 per group). ^*^*P* < 0.05 compared with the control group.

Figure S2 NKCC1 mRNA levels in MLE-12 cells. A nonsignificant decrease of NKCC1 mRNA expression was found in HR + bumetanide group in comparison to the HR group (*n* = 3 per group). ^*^*P* < 0.05 compared with the control group. BMT: bumetanide 20-μM. CTRL: control. Data are expressed as the means ± SD.

Figure S3 Expressions of p38 MAPK and α-ENaC in rats. **(A)** α-ENaC levels, **(B)** total p38 MAPK (T-p38) and phosphorylated p38 MAPK (p-p38) after IR treated with BMT (*n* = 5 per group). **(C)** α-ENaC expression after IR treated by p38 MAPK inhibitor, BIRB-796 0.3 mg/kg (*n* = 3 per group). BMT: bumetanide 70 μg/kg. CTRL: control. Data are expressed as the mean ± SD. ^*^*P* < 0.05 compared with the control group; ^#^*P* < 0.05 compared with the HR group.

Figure S4 Expressions of NF-κB and p38 MAPK in MLE-12 cells. **(A)** Control. Parameters were measured **(B)** 1 h, **(C)** 2 h, and **(D)** 4 h followed by reoxygenation (*n* = 3 per group). Bumetanide 20-μM had the better effect to suppress NF-κB and p-38 MAPK, and bumetanide 40-μM may have cytotoxic effect. Bumetanide effects to NF-κB and p-38 MAPK were similar followed by reoxygenation 1, 2, and 4 h. BMT: bumetanide. CTRL: control. Data are expressed as the mean ± SD. ^*^*P* < 0.05 compared with the control group. ^**^*P* < 0.01 compared with the control group.
