# Supplementary figures and images for: Inhibition of NKCC1 Modulates Alveolar Fluid Clearance and Inflammation in Ischemia-Reperfusion Lung Injury via TRAF6-Mediated Pathways
Source: Front Immunol. 2018 Sep 13;9:2049. doi: 10.3389/fimmu.2018.02049 (PMC6146090; doi:10.3389/fimmu.2018.02049)

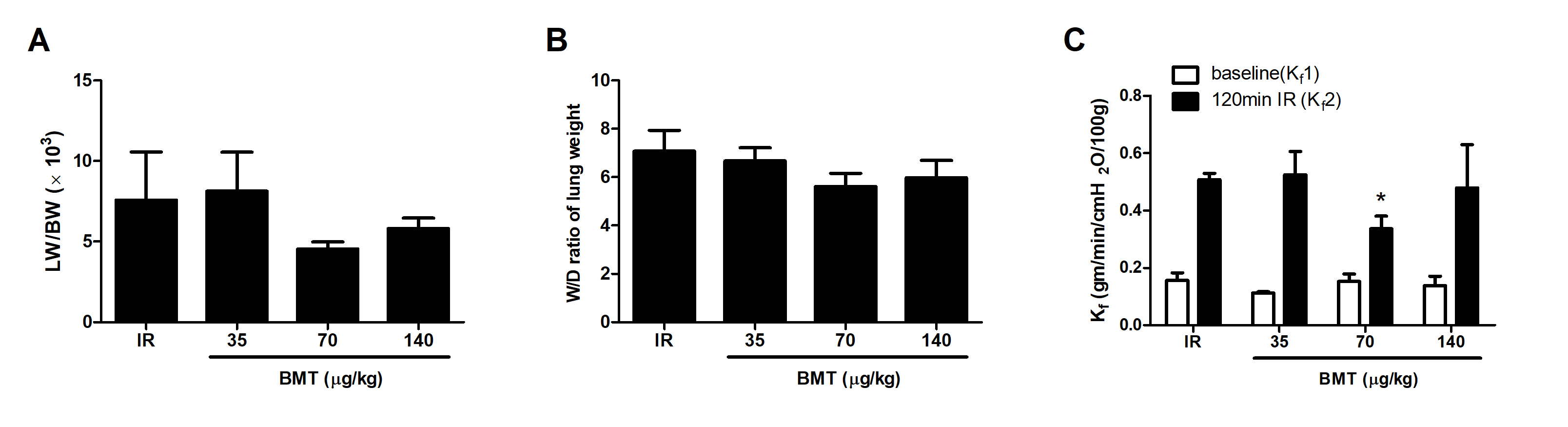

Supplement: Figure S1 — Effects of different doses of BMT on lung edema. (A) Lung weight/body weight (LW/BW), (B) lung wet/dry (W/D) weight ratios, and (C) pulmonary microvascular permeability (Kf). The increase of Kf in the ischemia–reperfusion (IR) group was significantly attenuated by treatment with BMT 70 μg/kg. BMT: bumetanide. CTRL: control. Data are expressed as the mean ± SD (n = 3 per group). *P < 0.05 compared with the control group. [file Image_1.JPEG]

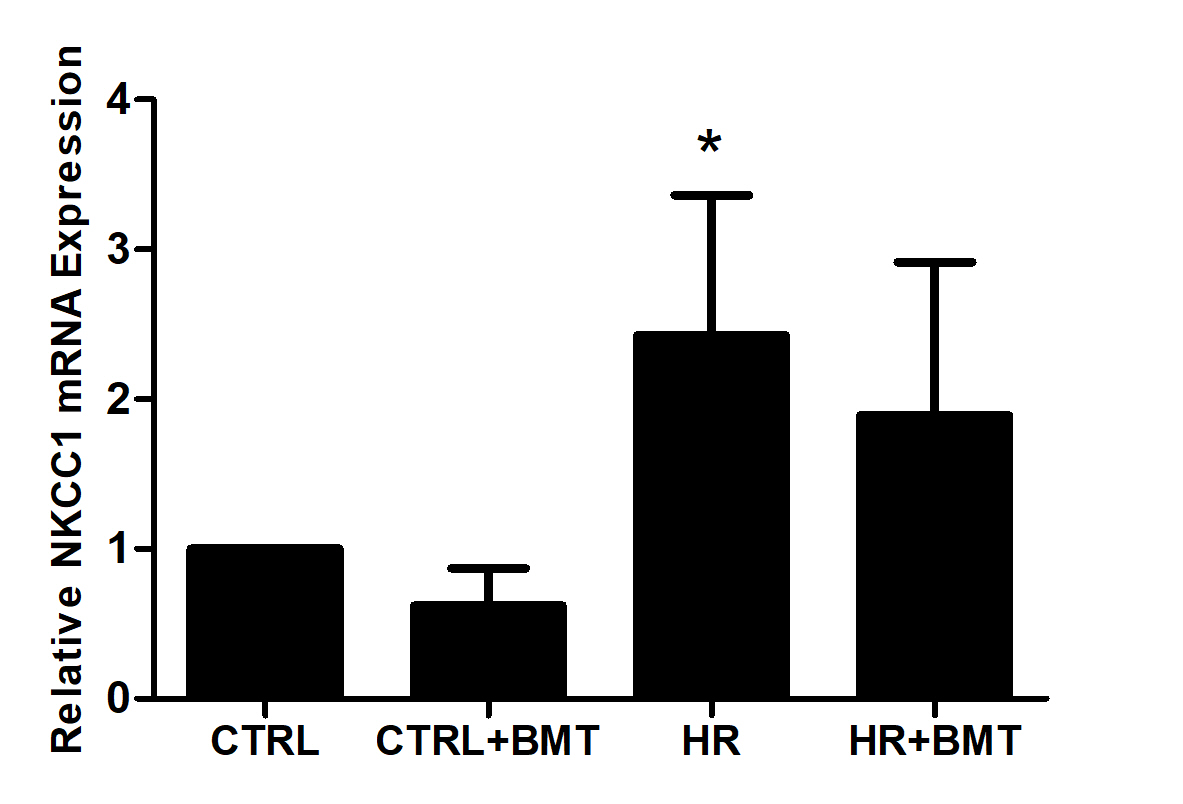

Supplement: Figure S2 — NKCC1 mRNA levels in MLE-12 cells. A nonsignificant decrease of NKCC1 mRNA expression was found in HR + bumetanide group in comparison to the HR group (n = 3 per group). *P < 0.05 compared with the control group. BMT: bumetanide 20-μM. CTRL: control. Data are expressed as the means ± SD. [file Image_2.JPEG]

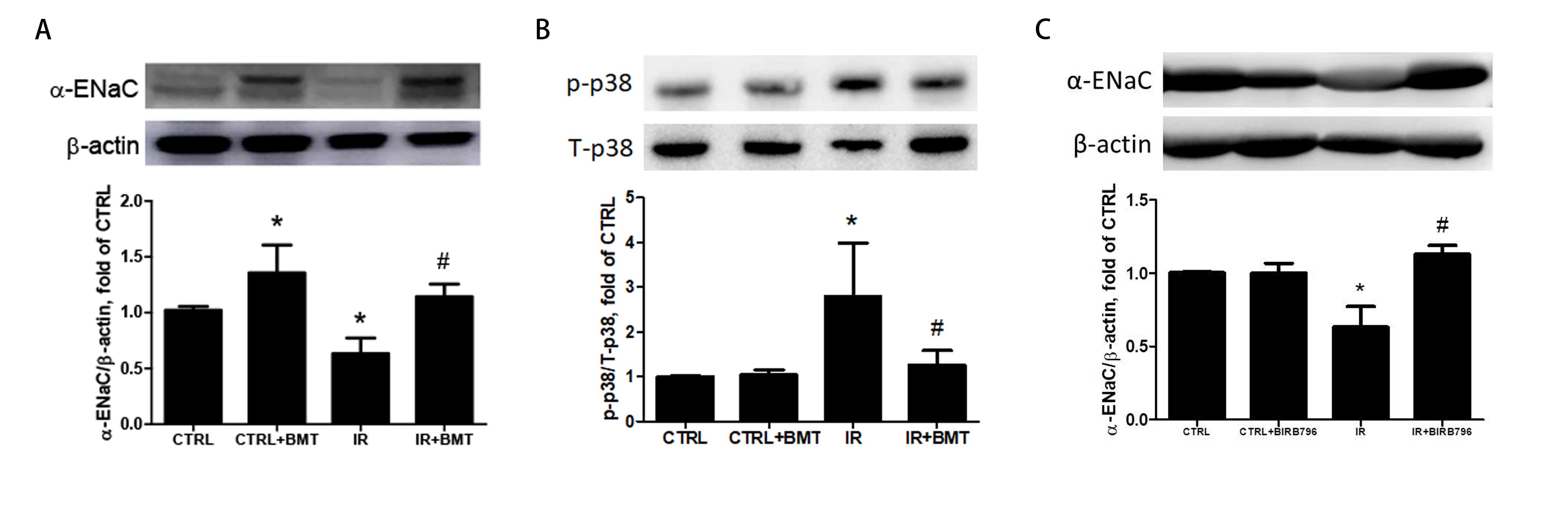

Supplement: Figure S3 — Expressions of p38 MAPK and α-ENaC in rats. (A) α-ENaC levels, (B) total p38 MAPK (T-p38) and phosphorylated p38 MAPK (p-p38) after IR treated with BMT (n = 5 per group). (C) α-ENaC expression after IR treated by p38 MAPK inhibitor, BIRB-796 0.3 mg/kg (n = 3 per group). BMT: bumetanide 70 μg/kg. CTRL: control. Data are expressed as the mean ± SD. *P < 0.05 compared with the control group; #P < 0.05 compared with the HR group. [file Image_3.JPEG]

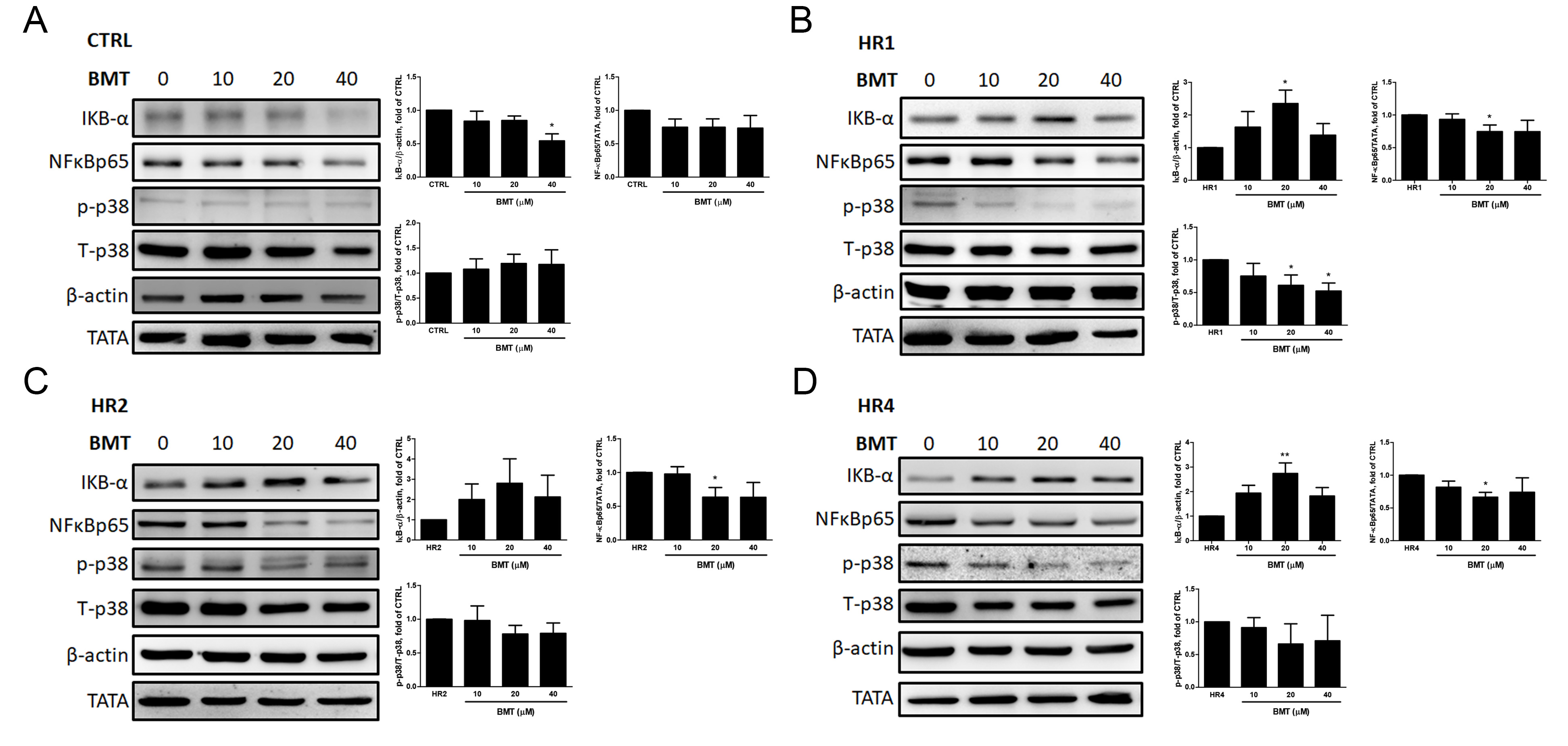

Supplement: Figure S4 — Expressions of NF-κB and p38 MAPK in MLE-12 cells. (A) Control. Parameters were measured (B) 1 h, (C) 2 h, and (D) 4 h followed by reoxygenation (n = 3 per group). Bumetanide 20-μM had the better effect to suppress NF-κB and p-38 MAPK, and bumetanide 40-μM may have cytotoxic effect. Bumetanide effects to NF-κB and p-38 MAPK were similar followed by reoxygenation 1, 2, and 4 h. BMT: bumetanide. CTRL: control. Data are expressed as the mean ± SD. *P < 0.05 compared with the control group. **P < 0.01 compared with the control group. [file Image_4.JPEG]
